# Molecular Dynamics Simulations Reveal Interactions of an IgG1 Antibody With Selected Fc Receptors

**DOI:** 10.3389/fchem.2021.705931

**Published:** 2021-07-02

**Authors:** Sebastjan Kralj, Milan Hodošček, Barbara Podobnik, Tanja Kunej, Urban Bren, Dušanka Janežič, Janez Konc

**Affiliations:** ^1^ Theory Department, National Institute of Chemistry, Ljubljana, Slovenia; ^2^ Laboratory of Physical Chemistry and Chemical Thermodynamics, Faculty of Chemistry and Chemical Engineering, University of Maribor, Maribor, Slovenia; ^3^ Biologics Technical Development Mengeš, Technical Research and Development Novartis, Lek Pharmaceuticals d.d., Mengeš, Slovenia; ^4^ Department of Animal Science, Biotechnical Faculty, University of Ljubljana, Ljubljana, Slovenia; ^5^ Faculty of Mathematics, Natural Sciences and Information Technologies, University of Primorska, Koper, Slovenia

**Keywords:** free energy calculation, homology modeling, molecular dynamics, fab-fcγ receptor interactions, monoclonal antibody, biological drugs

## Abstract

In a survey of novel interactions between an IgG1 antibody and different Fcγ receptors (FcγR), molecular dynamics simulations were performed of interactions of monoclonal antibody involved complexes with FcγRs. Free energy simulations were also performed of isolated wild-type and substituted Fc regions bound to FcγRs with the aim of assessing their relative binding affinities. Two different free energy calculation methods, Molecular Mechanical/Generalized Born Molecular Volume (MM/GBMV) and Bennett Acceptance Ratio (BAR), were used to evaluate the known effector substitution G236A that is known to selectively increase antibody dependent cellular phagocytosis. The obtained results for the MM/GBMV binding affinity between different FcγRs are in good agreement with previous experiments, and those obtained using the BAR method for the complete antibody and the Fc-FcγR simulations show increased affinity across all FcγRs when binding to the substituted antibody. The FcγRIIa, a key determinant of antibody agonistic efficacy, shows a 10-fold increase in binding affinity, which is also consistent with the published experimental results. Novel interactions between the Fab region of the antibody and the FcγRs were discovered with this *in silico* approach, and provide insights into the antibody-FcγR binding mechanism and show promise for future improvements of therapeutic antibodies for preclinical studies of biological drugs.

## Introduction

As therapeutic agents, monoclonal antibodies possess key advantages over small-molecule drugs. These include target specificity, lower toxicity profiles, longer serum half-life and multiple cytotoxic modes of action. This versatility has led to a valuation predicted to be $137–220 billion by the end of year 2020 for the antibody drug market. With this potential of antibodies, the pharmaceutical industry is searching for ways to improve existing therapies and cutting into the future market share (Grilo and Mantalaris, 2019). Currently, all FDA-approved therapeutic antibodies belong to the immunoglobulin isotype G (IgG) (Brezski and Georgiou, 2016), one of five isotypes of human antibodies or immunoglobulins (Franklin, 1975). The IgG antibody is a heterodimer consisting of two light chains and two heavy chains. The fragment antigen binding (Fab) domain is responsible for specific antigen recognition, while the C-terminal part of both heavy chains forms the fragment crystallizable (Fc) domain. This domain is responsible for immune effector functions associated with antibodies.

**FIGURE 1:**
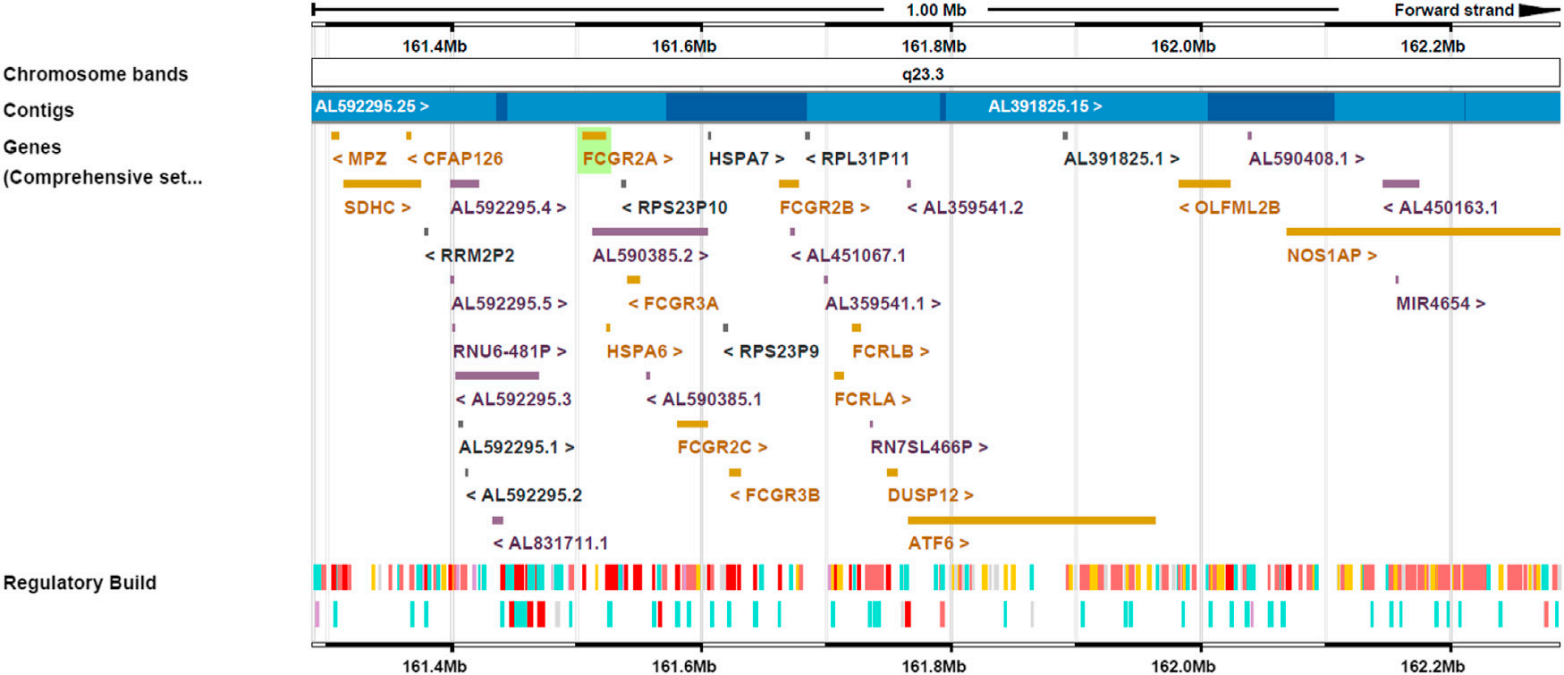
Genomic region of the low-affinity Fcγ receptor cluster on human chromosome 1q23.3. FCGR2A, FCGR2B and FCGR2C are located on the forward strand and FCGR3A and FCGR3B on the reverse strand.

The efficacy of many antibodies is associated with antibody dependent cellular cytotoxicity (ADCC), complement dependent cytotoxicity (CDC) and antibody dependent cellular phagocytosis (ADCP) to deplete target cells is mediated through interaction of the Fc region with the complementary component C1q or Fcγ receptors (FcγR). These are expressed in a broad spectrum of immune cells, and formation of an Fc/FcγR complex recruits these cells to sites of the bound antigen. The IgG antibodies predominantly elicit ADCC and ADCP by interacting with FcγRs. In humans, the FcγR protein family consists of FcγRI, FcγRII (subtypes a/b/c) and FcγRIII (subtypes a/b) (Raghavan and Bjorkman, 1996). All FcγRs bind the same region on the IgG Fc, with the FcγRI classified as a high affinity FcγRs and the FcγRII and FcγRIII as the low affinity FcγRs. The FcγRI, FcγRIIa/c and FcγRIIIa are activating receptors characterized by an intracellular immunoreceptor tyrosine-based activation motif, while the FcγRIIb is an inhibitory receptor characterized by an inhibition motif (Maenaka et al., 2001) (Table 1). The genomic region of the low-affinity Fcγ receptor cluster on human chromosome 1q23.3 is presented in Figure 1.

**TABLE 1:** Characteristics of the FcγRs. Data extracted from the HGNC database (https://www.genenames.org/).

	Affinity	Function	Gene symbol	Gene name	Gene HGNC ID	Chromosomal location	Alias symbols
FcγRIa	High	Activating	*FCGR1A*	Fc fragment of IgG receptor Ia	3,613	1q21.2	CD64, CD64A
FcγRIb	High	Activating	*FCGR1B*	Fc fragment of IgG receptor Ib	3,614	1p11.2	CD64b
FcγRIIa	Low	Activating	*FCGR2A*	Fc fragment of IgG receptor IIa	3,616	1q23.3	CD32, CD32A
FcγRIIb	Low	Inhibiting	*FCGR2B*	Fc fragment of IgG receptor IIb	3,618	1q23.3	CD32, CD32B
FcγRIIc	Low	Activating	*FCGR2C*	Fc fragment of IgG receptor IIc	15,626	1q23.3	hFcRII-C, D32C
FcγRIIIa	Low	Activating	*FCGR3A*	Fc fragment of IgG receptor IIIa	3,619	1q23.3	CD16, CD16a
FcγRIIIb	Low	Activating	*FCGR3B*	Fc fragment of IgG receptor IIIb	3,620	1q23.3	CD16, CD16b

Antibodies possess multiple cytotoxic modes of action, but many have failed in clinical trials due to insufficient efficacy. This has led to attempts to increase their potency through enhancement of their ability to mediate cellular cytotoxicity functions such as ADCC and ADCP (Weiner and Carter, 2005). Further studies have found the Fc region to be essential for the therapeutic efficacy of antibodies that rely on ADCC or ADCP (Clynes et al., 2000; Arce Vargas et al., 2018; Lešnik et al., 2020). In order to achieve optimal therapeutic efficacy, specific sub-types of FcγR must engage with the Fc region. Increased binding to FcγRIIa or FcγRIIIa results in greater ADCP and ADCC activity, a desirable effect in many therapeutic antibodies, but an increase in FcγRIIb binding is desired for inhibitory antibodies. Achieving this is difficult as FcγRIIb and FcγRIIa demonstrate ∼92% homology in their extracellular domains despite the fact that they differ functionally. However, several successful engineered Fc variants with increased binding affinity to human FcγRIIIa have been reported (Lazar et al., 2006; Richards et al., 2008; Liu et al., 2014; Wang et al., 2018). These variants include the single mutants S239D and I332E, the double mutant S239D/I332E, and the triple mutant S239D/I332E/A330L (Liu et al., 2014). All of these variants have also been linked to enhanced ADCC activity (Lazar et al., 2006). On the spectrum of increasing FcγRIIa binding and enhancing phagocytosis, the reported Fc variant G236A has selectively enhanced binding to FcγRIIa compared to FcγRIIb and mediates enhanced phagocytosis of antibody-coated target cells by macrophages (Richards et al., 2008; Wang et al., 2018).

We have examined the molecular dynamics of both the complete antibody structures and the isolated Fc region and have conducted binding free energy calculations to gain insight into their interactions with various FcγRs and to obtain directions which could lead to improved antibodies. We used homology modeling (Šali and Blundell, 1993) to obtain a new structure of a complete therapeutic IgG1 antibody, to which we examined the binding of FcγRIIa, FcγRIIb and FcγRIIIa using existing structures from the Protein Data Bank (PDB). Using the CHARMM biomolecular simulation program (Brooks et al., 2009) we performed 100 ns simulations for both the wild type (wt) antibody and the antibody with the G236A substitution in the lower hinge of the Fc region, a substitution known to increase the ADCP selectively (Richards et al., 2008). The calculated free energy values for the complete antibody simulations agree with the published experimental results (Richards et al., 2008), which show that FcγRIIIa enjoys a higher affinity than both FcγRIIa and FcγRIIb, with FcγRIIa having a higher affinity than FcγRIIb. However, the wild type and substituted antibody free energy values are not entirely in agreement with the published results for complete antibodies (Richards et al., 2008). The relatively higher agreement of the Fc-FcγR simulations with the experimental data is probably due to the negation of the effects of Fab-FcγR interactions seen in these simulations. With the more stringent conditions of the BAR free energy calculation method, a large increase in affinity is observed exclusively for FcγRIIa, as has been reported by Richards (Richards et al., 2008). The agreement of the calculated energies with the experimental data lends credibility to the *in silico* approach to future prospective evaluations of potential effector substitutions.

## Methods

### Homology Modeling of the IgG1 Therapeutic Antibody and Preparation of Structures

The sequence of the therapeutic IgG1 antibody in the FASTA format was obtained from the DrugBank database (https://www.drugbank.ca, accessed on date August 28, 2019). Structural templates for this sequence were found using the blastp algorithm (Altschul et al., 1990) with default settings. Only similar sequences found in the PDB were retained. The templates were selected based on sequence identity, query cover and resolution of the solved structure. For modeling of the heavy chain of a therapeutic antibody we used the structure of an intact human IgG1 (PDB ID:1HZH) (Saphire, 2001) which has 100% query cover and 84% sequence identity to the target sequence. 1HZH, a complete IgG1 antibody structure, enabled us to correctly spatially orient the Fab arms in our model. As monoclonal antibodies differ significantly in the Fab variable region which has both the light and heavy chains, an additional template was used to improve the quality of the model in this region. The structure of ipilimumab bound to the human receptor CTLA-4 (PDB ID:5TRU) was selected based on the 100% query cover and 100% sequence identity to the light chain of the investigated therapeutic antibody (Ramagopal et al., 2017). High query cover and sequence identity are crucial for the final quality of the obtained homology model. Templates were aligned to their corresponding targets using the MUSCLE sequence alignment algorithm (Edgar, 2004). The alignment was checked for potential gaps or misaligned residues before the modeling. Protein models were constructed using the MODELLER software, builds a model of the protein by satisfying all spatial restraints (Šali and Blundell, 1993). The models obtained in this way were evaluated using the discrete optimized protein energy (DOPE) score. The lowest scoring model, with a DOPE value of −131,997 was further checked for quality using several homology model validating tools (see Supplementary Material) and was chosen for further work (Shen and Sali, 2006).

**FIGURE 2:**
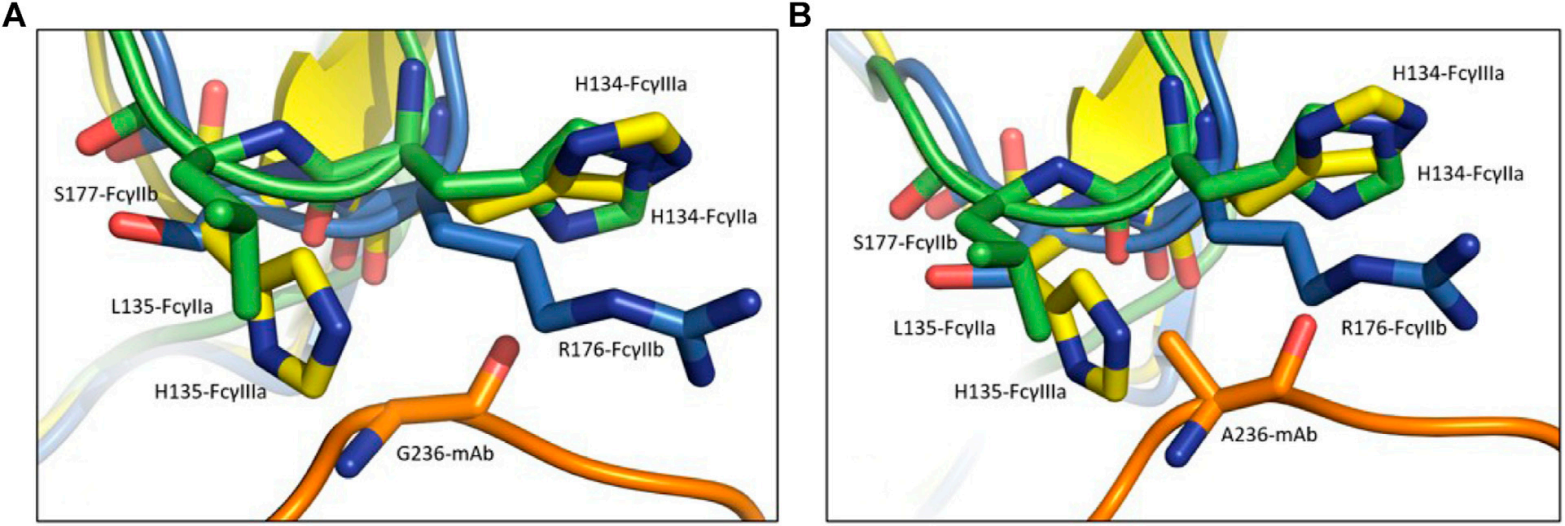
The differences between the FcγRs were exploited to selectively enhance binding of the antibody to the activating Fcγ receptors. The effector substitution G236A is in the Fc region of the antibody (orange) in the complex with the inhibitory FcγRIIb (blue), and the activating FcγRIIa (green) and FcγRIIIa (yellow). In comparison to the wild-type Gly236 **(A)**, the substituted Ala236 **(B)** is bulkier thus preventing the binding of the antibody to the FcγRIIb due to the steric clash with Arg176, while allowing the binding to FcγRIIa and FcγRIIIa that have histidines at this position.

Models of the selected FcγRs complexed with the complete antibody structure were generated (Figure 2). The criteria for the selection of FcγR structures from the PDB were the resolution of the solved structure and the scope of the FcγR glycosylation profile, since glycosylation of both the Fc region and the FcγR play a crucial role in the binding mechanics of these two proteins (Hayes et al., 2014). The PDB structure of the FcγRIIIa bound to antibody Fc region (PDB ID:3SGK) contains the intact IgG1 Fc region bound to FcγRIIIa (Ferrara et al., 2011). The Fc region in the PDB structure 3SGK was superimposed on the modeled complete therapeutic IgG1 antibody to position the FcγRIIIa so that it was bound to our model. The redundant Fc region of 3SGK crystal structure was deleted to produce the final model of the antibody in a complex with FcγRIIIa. The other two models were then constructed by superimposing the structures of FcγRIIa (1FCG) and FcγRIIb (5OCC) onto the correctly positioned antibody (Hogarth et al., 1999; Sutton et al., 2018). This was possible because the selected FcγRs, FcγRIIa, FcγRIIb and FcγRIIIa bind to the same region of the IgG1 Fc region and in a similar conformation. Structural files for each of the FcγRs bound to the antibody were saved as PDB files to be used as inputs for molecular dynamics. For the isolated Fc regions, no homology modeling was performed. The PDB structure 3SGJ was chosen because of its extended hinge region which was not expected to interfere with the receptors during the simulation (Ferrara et al., 2011). The same three FcγRs used for the complete antibody simulations, were superimposed to the structure and saved as separate files.

### Molecular Dynamics Simulations of IgG1-FcγR Complexes

We performed MD simulations of the complete IgG1 therapeutic antibody with selected FcγRs and simulations of the isolated Fc region with various bound FcγRs, using the latter to calculate the impact of substitution on binding free energy. Finally, we compared the *in silico* results obtained with experimental binding affinities. The use of MD simulations allows us to study the motion of our system through time. This is achieved by numerically integrating Newton’s second law of motion. The simulation inputs were prepared using the CHARMM-GUI web interface for the CHARMM biomolecular simulations program (Jo et al., 2008; Brooks et al., 2009). CHARMM-GUI Glycan Modeler was used to apply the most prevalent experimentally determined human glycosylation profile: {bDGal (14)bDGlcNAc(1→2)aDMan (1→6)[bDGlcNAc(1→2)-aDMan (1→3)] bDMan (1→4) bDGlcNAc(1→4)[aLFuc (1→6)]bDGlcNAc(1→)ASN-297)} for IgG1 antibodies based on experimental data (Sonneveld et al., 2018), as the homology model PDB structure was unglycosylated (Park et al., 2019). The protein structures were solvated using TIP3P water, and then neutralized using Na^+^ and Cl^−^ ions (0.1 M) to approximate physiological conditions. For the removal of steric clashes, that could be present after merging coordinate files of water molecules with proteins and, to optimize atomic coordinates of the complexes, 50 steps of steepest descent and 250 steps of adopted basis Newton-Raphson (ABNR) energy minimizations were performed. Both functions attempt to minimize the potential energy of the system, by slightly nudging the atomic coordinates of the protein followed by potential energy calculation and examination of the first derivatives to determine the direction of the gradient. Nudges of coordinates which result in lower potential energy i.e. moving towards a local minimum are saved, and the process is repeated with new coordinates until the specified step number.

This is followed by a short MD simulation during which the protein was equilibrated at 310.15 K using the HOOVER thermostat and the integration time-step set to 1 fs The total length of equilibration molecular dynamics with NVT ensemble applied was 1 ns Final molecular dynamics production runs were carried out using an NPT ensemble with periodic boundary conditions applied, the time-step set to 2 fs and the HOOVER thermostat set to 310.15 K. Van der Waals interactions were cutoff between 10 and 12 Å using the force switch method (VFSWIt). Electrostatic potential used force shifting method (FSHIft) with a cutoff of 12 Å. The particle-mesh Ewald summation (Darden et al., 1999) was used to calculate electrostatic interactions. Bonds to hydrogens were constrained using the SHAKE algorithm. This allows for a 1–2 fs integration step as otherwise unconstrained hydrogens, which have high frequency vibrating bonds lead to errors when integrating Newton’s second law of motion. The force field, a simplified representation of reality allows us to derive the forces required for solving Newton’s second law. The CHARMM36m force field was used for all simulations (Brooks et al., 2009; Guvench et al., 2011). For each of the selected FcγRs bound to either the complete antibody structure or the isolated Fc region production runs were generated using GPU acceleration with the final analysis performed on the last 100 ns for the complete antibodies and 200 ns for the isolated Fc region. In order to solve Newton’s second law of motion velocities of atoms beside forces are required as well. Velocities are randomly generated at the start of the simulations. For this reason the first 20 ns of production runs were ignored to minimize the error arising from different initial velocities, as the additional time before sampling allows the protein to settle.

### Calculation of the Binding Free Energy

The final result of MD simulations is the trajectory file that contains the information of how the protein moved in time. Beside visual cues that are offered from this file, we can calculate thermodynamic properties from it. In this paper two different approaches were used to calculate the relative binding free energies for the simulated complexes, the end-point Molecular Mechanical/Generalized Born molecular volume (MM/GBMV) method and the Bennett Acceptance Ratio (BAR) method (Bennett, 1976). The relative binding free energy was calculated, rather than the absolute binding free energy, since calculation of absolute binding free energies for biological events requires much longer simulations.

**FIGURE 3:**
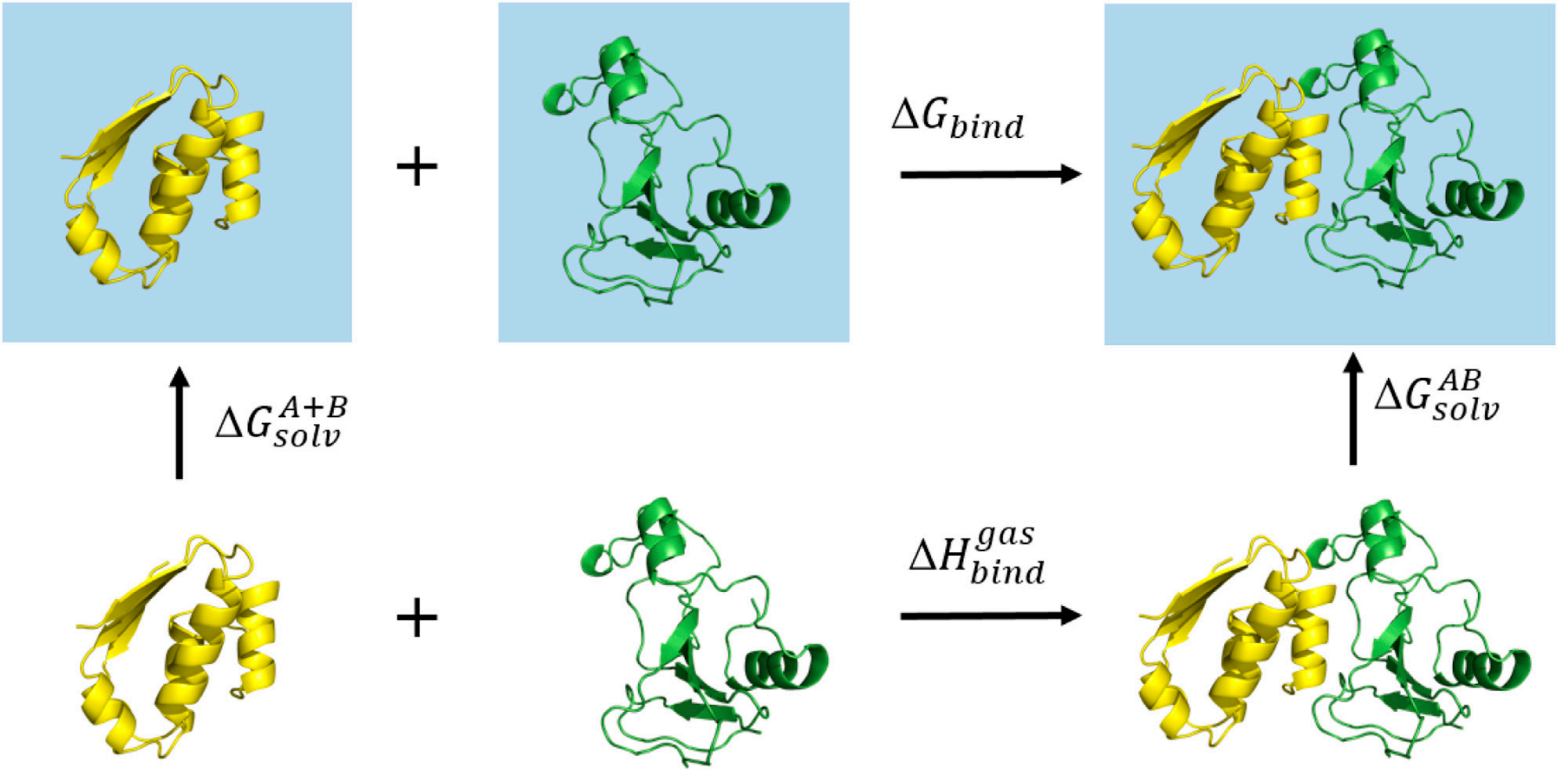
The thermodynamic cycle used in the MM/GBMV calculation. The square blue surface represents the water solvent.

The MM/GBMV(Lee et al., 2002) method implemented in CHARMM decomposes the free energy of binding of the ligand, the protein and the complex, into contributions of different interactions (Figure 3) and can be expressed as follows:

$$\Delta {\text{G}}_{\text{bind}}\;=\;\Delta \text{H }-\text{ T}\cdot \Delta \text{S}\approx \Delta {\text{E}}_{\text{MM}}+\Delta {\text{G}}_{\text{sol}}\;-\text{ T}\cdot \Delta \text{S}$$ (1)

$$\Delta {\text{E}}_{\text{MM}}=\;\Delta {\text{E}}_{\text{int}}+\Delta {\text{E}}_{\text{el}-\text{st}}+\Delta {\text{E}}_{\text{vdw}}$$ (2)

$$\Delta {\text{G}}_{\text{sol}}=\;\Delta {\text{G}}_{\text{generalized}-\text{Born}}+\Delta {\text{G}}_{\text{molecular volume}}$$ (3)

In these equations, ΔEMM represents the changes in the gas-phase molecular mechanics energy and includes changes in the internal energy (ΔEint) (bond, angle and dihedral), electrostatic (ΔEel-st) and the van der Waals energy (ΔEvdw). The effect between the solute and the implicit solvent is described by ΔGsol, which represents the sum of the polar and non-polar contribution to the desolvation free energy with the polar contribution (ΔGgeneralized-Born) calculated by the Generalized Born using Molecular Volume (GBMV) model implemented in CHARMM and the molecular volume contribution (ΔGmolecular volume) estimated by the molecular volume calculation implemented within the GB module (Lee et al., 2002, 2003). The entropic contribution due to vibrational modes of the system to the binding free energy was neglected as the aim was to calculate relative binding free energies. Furthermore, the calculation of the entropic contribution of protein-ligand binding is only relevant in binding events where large conformational changes occur. Such conformational changes were not expected in this case, as we had very similar complexes (wild type and G236A), and consequently we neglected the entropy term (Konc et al., 2013). For calculation of free energy using the MM/GBMV approach a single MD simulation of a protein-ligand complex was used, from which we obtained three separate trajectories of all components, the ligand, the apo protein and the protein-ligand complex (Lee and Olson, 2006). For the complete antibody simulations, all energy terms with the exception of the entropy terms were calculated for the final 100 ns(10,000 snapshots) of the production run for each MD trajectory. The energy terms for the isolated Fc region with the bound FcγR were calculated for 200 ns(20,000 snapshots).

**FIGURE 4:**
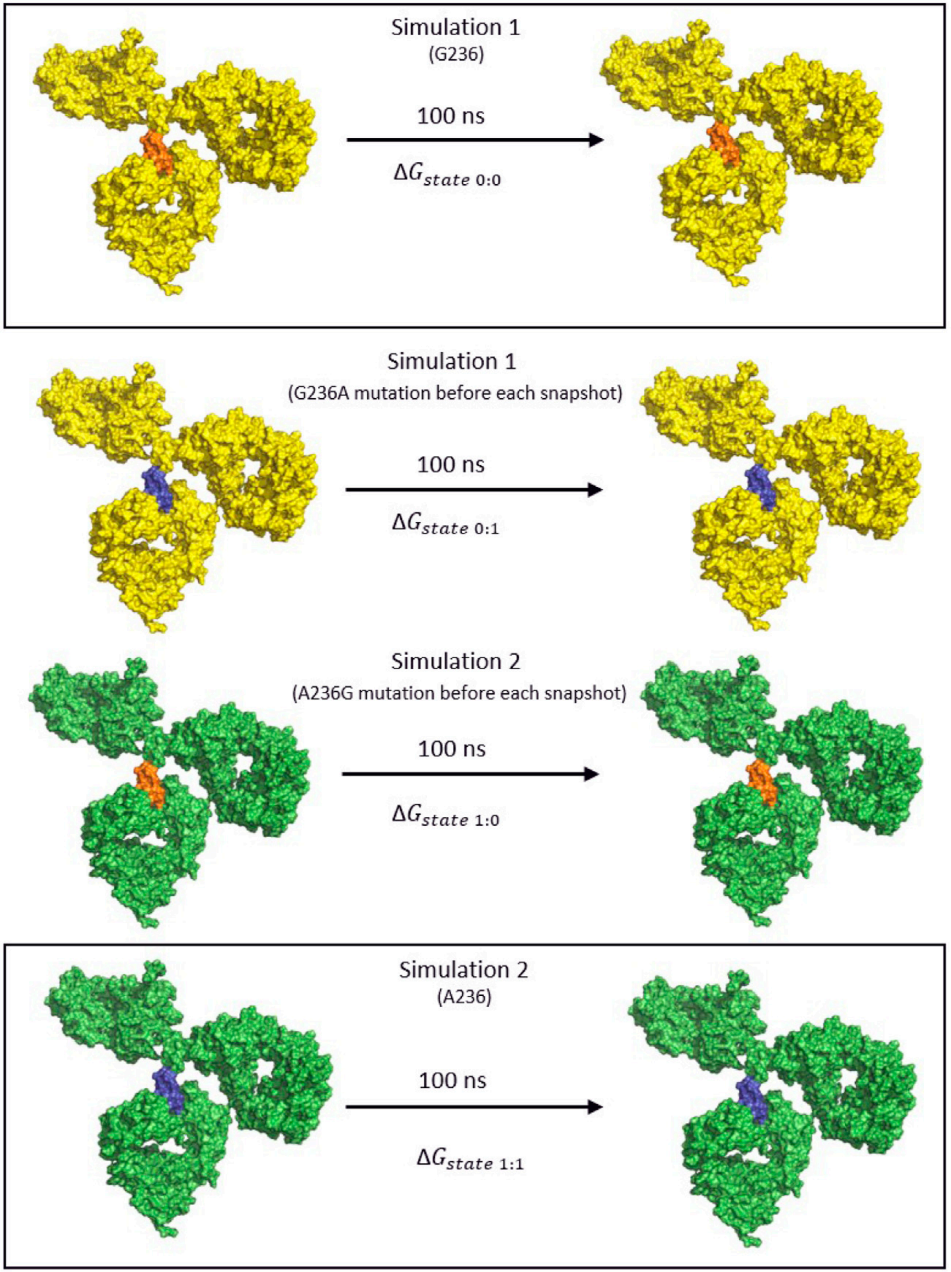
The thermodynamic cycle for the calculation of the binding free energy using the BAR method. The IgG1 antibody is colored yellow for the first simulation and green for the second, the Gly236 variant is orange and the mutated Ala236 is blue. Two simulations were performed for each of the FcγR bound to the antibody. Subsequently, the intermediate trajectories 0→1 and 1→0 were obtained by mutating the original amino acid of the given simulation at every snapshot of the simulation before calculating the free energy terms.

The Bennett Acceptance Ratio (BAR) method is a rigorous method for calculating relative binding free energy but it offers greater accuracy (Bennett, 1976). Using the coupling parameter, λ to define intermediate states, it calculates the free energy difference between end-states A and B. This is useful as two similar systems, such as a wild type and substituted antibody can show very little overlap in phase space, making estimation of the free energy difficult when relying solely on the end states. In the production runs that were obtained, each snapshot of the simulation was submitted to an energy minimization of 100 steps of ABNR or until the specified tolerance (tolgrd) was 1.0 before calculating the energy. This was done to prevent steric clashes that might arise when inserting (mutating) the amino-acid into the non-mutated simulation snapshot. In the case of glycine to alanine the presence of the additional –CH3 after mutation might cause steric clashes with the amino acids in the vicinity, giving false energy calculations. The non mutated simulation paths (0:0,1:1, Figure 4) were minimized as well to achieve consistency of energies. The energy data was filtered using an in-house python script, and the average ΔΔG differences for the simulations were obtained using the BAR script for CHARMM. No additional intermediate *λ* states were defined, as great overlap between the phase space of both simulations was achieved using only the end states (Figure 4).

## Results and Discussion

### Free Energy Calculations of the Effector G236A Substitution

**FIGURE 5:**
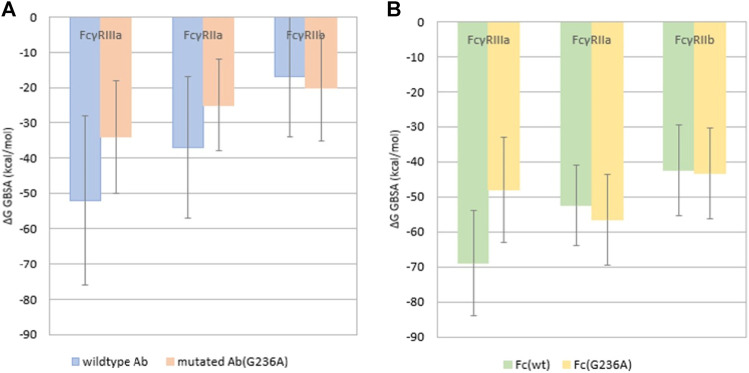
Binding free energy calculated using the MM/GBMV method for the complex of **(A)** the complete antibody with FcγRs and **(B)** the isolated Fc region with FcγRs. The error bars represent standard deviations.

The effect of the G236A substitution on the binding affinity of the monoclonal antibody with different Fcγ receptors (FcγRs) was assessed by the free energy MM/GBMV and BAR methods using the simulations of the complete antibody as well as its isolated Fc region. The calculated binding affinities are in agreement with the experiment by Richards et al. using the MM/GBMV method; in both simulations, FcγRIIIa exhibits the highest overall affinity of the three receptors, FcγRIIb the lowest, while FcγRIIa is intermediate (Figure 5).

It was also reported (Richards et al., 2008) that the G236A substitution in the Fc region of the antibody results in selective increase of the binding of FcγRIIa over that of FcγRIIb or FcγRIIIa. The complete antibody simulations do not confirm this (Figure 5A), as the calculated binding free energy of the wild type antibody in complex with FcγRIIa is −36.9 ± 20 kcal/mol, but increases to −25.2 ± 13 kcal/mol for the mutated antibody. This indicates that the G236A substitution decreases the binding affinity. For the FcγRIIIa wild type antibody the binding affinity drops significantly, as the binding free energy for the wild type antibody is −52.1 ± 24 kcal/mol and for the substituted it is −33.8 ± 16 kcal/mol. When comparing the number of hydrogen bonds formed between the two antibodies (wild type and Ala236) bound to FcγRIIIa, the Ala236 antibody surprisingly forms more hydrogen bonds, on average 11.49 for the Ala236 antibody, and 4.85 for the wild type antibody. This difference could be due to different types of hydrogen bonds, with variable strengths. The binding affinity of the complete antibody towards FcγRIIb increases slightly from −17.4 ± 17 to −20 ± 15 kcal/mol, but the number of hydrogen bonds formed decreases from 4.79 (78%) in the wild type compared to 2.11 (32%) in the Ala236 antibody, suggesting a decrease in the binding affinity. When comparing the wild type antibody with the G236A substituted antibody we were unable to establish a clear correlation with the reference experimental values.

It was observed that the trajectories of the complete antibodies contained, in addition to the expected interactions between the Fc regions and the FcγRs, many interactions between the Fab regions and the FcγRs. It is thought that these Fab interactions may have disrupted the Fc-FcγRs interactions that were studied. This can be seen by comparison of Figures 5A,B, which shows that the binding affinity for the isolated Fc region (Figure 5B) is higher in all cases than the binding affinity of the complete antibody simulations (Figure 5A). This higher affinity for isolated Fc regions is thought to be due to the destabilizing effects of the Fab-FcγRs interactions that were seen with the complete antibody simulations, which may reduce the binding of the FcγRs with the complete antibody.

To examine the possible effects of the Fab region on the binding affinity we performed additional simulations using the isolated Fc regions in complexes with FcγRs (Figure 5B). Here, in accordance with existing data (Richards et al., 2008), the FcγRIIa exhibits a ∼4 kcal/mol decrease in binding free energy, consequently an increase in binding affinity after introducing the G236A substitution, which drops from −53.2 ± 11 kcal/mol to −57.1 kcal/mol. The FcγRIIIa shows an increase in binding free energy, or a decrease in binding affinity after substitution, probably due to the steric clash of the His135 in FcγRIIIa with the Ala236 methyl group (Figure 2). The decrease in the binding affinity for FcγRIIIa is −21 kcal/mol, from −68.9 ± 15 kcal/mol for the wild type Fc region to −48 ± 15 kcal/mol for the Ala236 substituted Fc region. The FcγRIIb shows no significant increase in binding free energy, which goes from −42.4 ± 13 kcal/mol for the wild type Fc region to −43.3 ± 13 kcal/mol for the Ala236 substituted Fc region, indicating no effect of this substitution on binding affinity. These findings are in agreement with experimental results (Richards et al., 2008).

**FIGURE 6:**
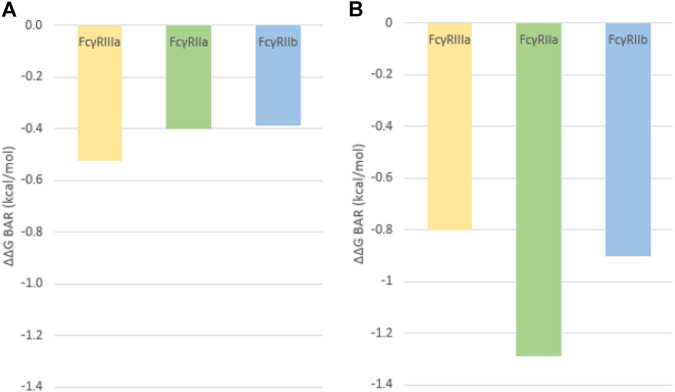
Relative binding free energies calculated using the BAR method and representing the relative change in binding free energy after the induction of the G236A substitution for **(A)** the complete antibody simulation and **(B)** the isolated Fc region simulation.

Results from the BAR method show that in fact the G236A substitution causes an increase of the binding affinity for all FcγRs (Figure 6). The effect of the Fab region on the FcγR binding is clearly visible as the simulation of the isolated Fc region shows a larger increase in binding affinity compared to the simulations of the complete antibody (*cf* Figures 6A,B). The value for the FcγRIIa is in alignment with published data (Richards et al., 2008) as the binding free energy increase of −1,29 kcal/mol indicates an almost 10-fold increase in the binding affinity. The binding affinity increases along all of the receptors when the substitution is present, but the largest increase for the Fc-FcγR simulations is seen in the activation of FcγRIIa, an important mediator of ADCP, compared to the inhibitory FcγRIIb and ADCC stimulating FcγRIIIa (Figure 6B). This indicates that introduction of this substitution strengthens the binding to FcγRIIa, resulting in a higher activation of macrophages (ADCP) and a better therapeutic outcome, a result that has been observed experimentally (Richards et al., 2008).

### Novel Interactions Between the FcγRs and the Fab Regions of the Therapeutic Antibody

**FIGURE 7:**
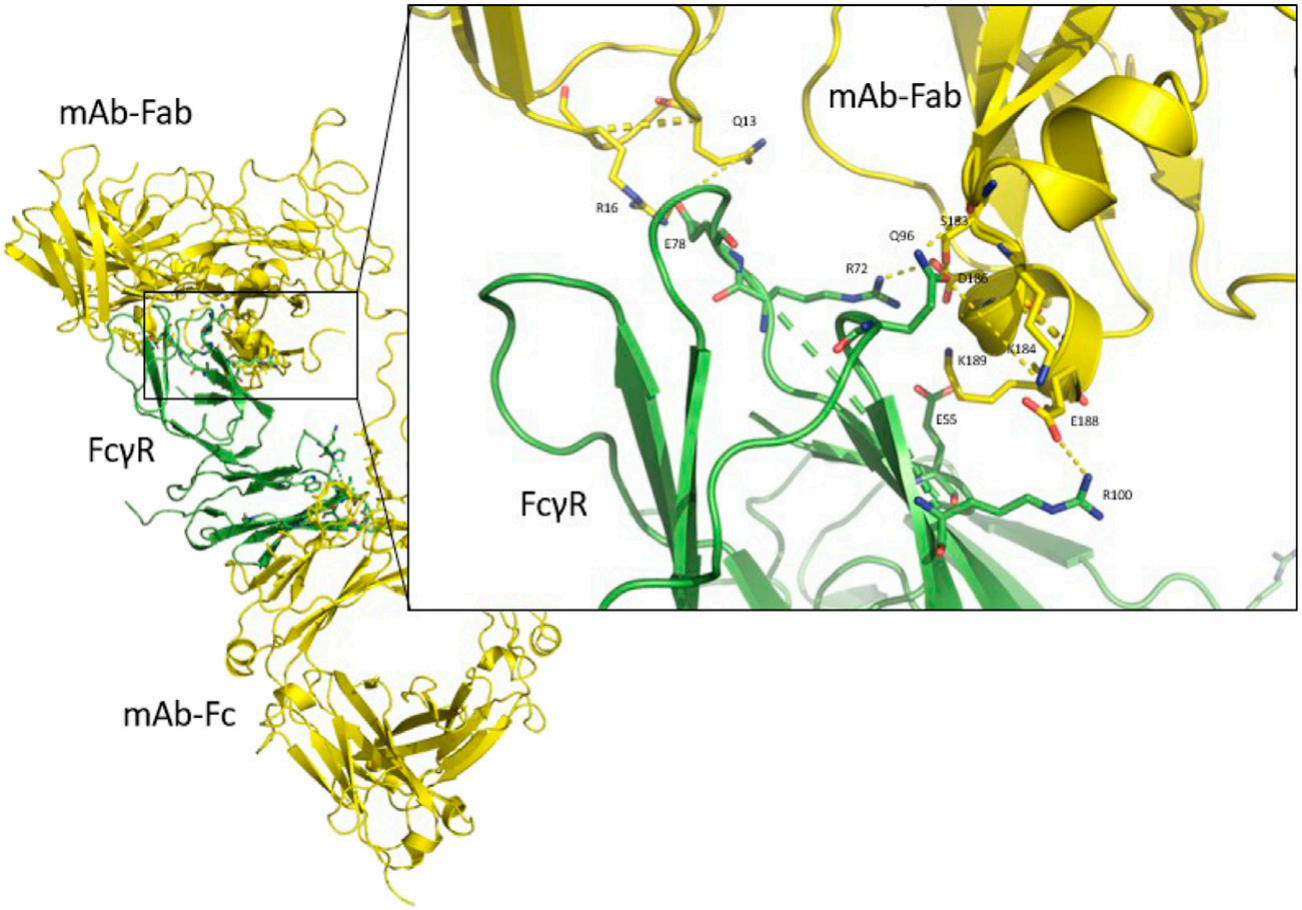
The complete antibody structure (yellow) interacting with FcγR (green) with both the Fc region and the Fab region. Close-up of the interaction between the Fab region, which interacts predominantly with its α-helix (residues 183–190), and the FcγR. Hydrogen bond interactions are shown as yellow dashed lines.

Upon inspection of the obtained trajectories of all the FcγRIIa, FcγRIIIa and FcγRIIb FcγRs, we discovered novel interactions of the Fab region of the therapeutic antibody with the selected FcγRs (Figure 7). This was unexpected as it is generally thought that interactions between an antibody and FcγRs occur exclusively in the Fc region of the antibody (Lu et al., 2015). The FcγR-Fab interactions observed in the simulations may be present *in vivo* and may play a previously unrecognized role in the binding of antibodies to FcγRs (Hogarth et al., 1999). To date, one study [see Figure 6 in Ref (Yogo et al., 2019)] reports such interactions with the receptor FcγRIIIa, and similar interactions were observed using MD simulations with the FcγRI (Zhao et al., 2019). Our simulations show that antibodies can interact similarly with FcγRs through their Fab regions, confirming this previous report (Yogo et al., 2019).

Our simulations show that the CH1 domain of the antibody’s Fab region forms the strongest interactions with the FcγR through an α-helix (residues 183–190) of the Fab light chain, which interacts with the upper region of the receptor (Figure 7, close-up). In addition, the loop (residues 12–17) of the heavy chain CH1 is also seen to interact with the FcγR, as has been observed experimentally (Yogo et al., 2019).

**FIGURE 8:**
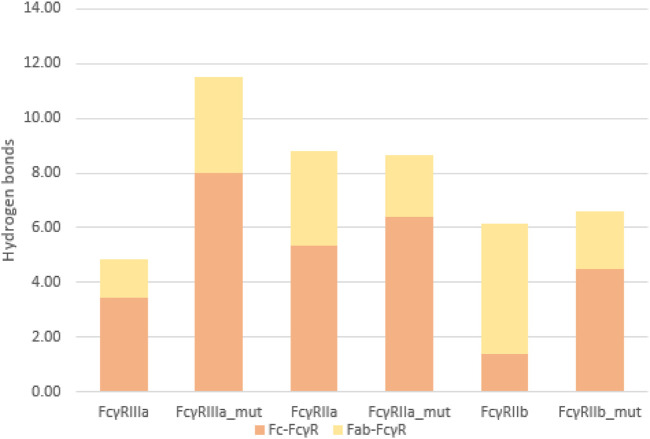
Average number of hydrogen bonds formed between the FcγR and the antibody during simulation: comparison between the Fab-FcγR (yellow) interactions and the Fc-FcγR interactions (orange).

**FIGURE 9:**
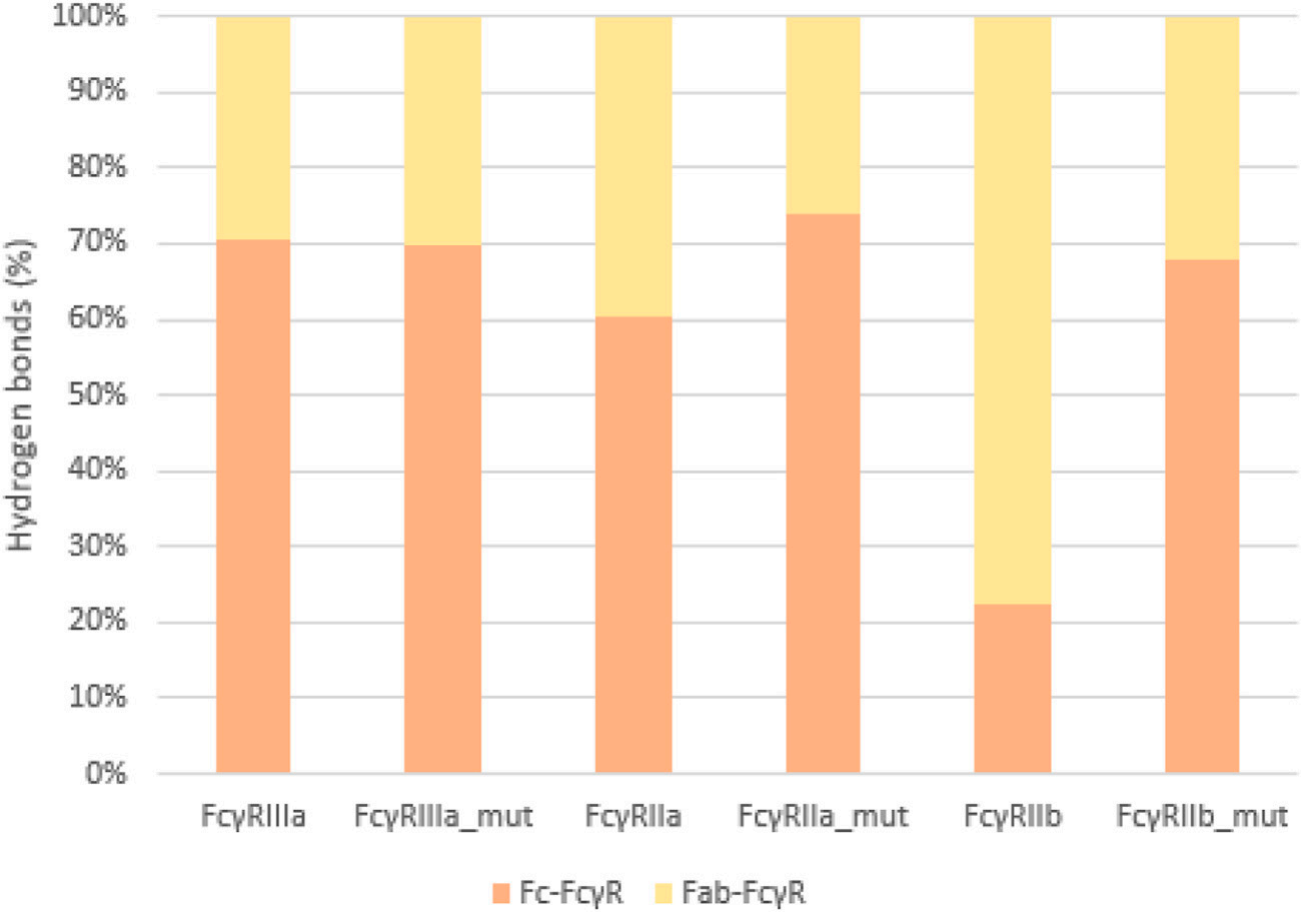
Hydrogen bonds as percentage of all interactions. Comparison between Fc-FcγR (orange) and Fab-FcγR (yellow) interactions shows that hydrogen bonds formed between Fab region and FcγR represent at least 30% of the interactions, indicating their relevance.

To quantify the interactions between the antibody and FcγRs, we calculated the average number of hydrogen bonds formed during simulations between the individual regions (Fc or Fab) and the FcγRs (Figure 8). The significant percentage of hydrogen bonds between the Fab region and the FcγRs (Figure 9) indicates that the Fab region may indeed play an important role in binding of the antibody to the FcγRs. Particularly, the wild type antibody in complex with FcγRIIb had predominantly the Fab region binding with the receptor, as in this complex, and the bonds between the Fab and the FcγR bonds amounted to 77% of all hydrogen bonds formed between the antibody and the FcγR. Across all the simulated complexes, the average proportion of the Fab-FcγR hydrogen bonds is 38% or 2.7 hydrogen bonds. These newly discovered hydrogen bond interactions reaffirm that the Fab region influences the binding of FcγRs, which has been reported in just one study to date (Yogo et al., 2019). Further, the comparison of the calculated binding free energies (Figure 5) and the hydrogen bond interactions (Figure 8) suggests that the interactions formed between the Fab region and FcγRs decrease the binding affinity of the Fc region for the receptors. This decrease in affinity however, is partially compensated for by the formation of new hydrogen bonds with the Fab region.

## Conclusions

Using the free energy simulation methods MM/GBMV and BAR, we have shown that the known substitution (G236A) has a selective effect on the binding of the antibody with FcγRs. The substitution increases the binding with FcγRIIa, thereby increasing ADCP, and to a lesser extent it increases binding affinity for the inhibitory FcγRIIb and the ADCC-activating FcγRIIIa, consistent with experiments (Richards et al., 2008). Through simulations of the complete antibody, we found novel interactions between the Fab region of the antibody and the FcγRs, which were experimentally determined recently using high-speed atomic force microscopy (Yogo et al., 2019). Our simulation results should be very valuable for future improvement of therapeutic monoclonal antibodies and could contribute to the development of new antibody therapeutic approaches.

## Data Availability

The original contributions presented in the study are included in the article/Supplementary Material, further inquiries can be directed to the corresponding authors.
