# Supplementary material for: Molecular Dynamics Simulations Reveal Interactions of an IgG1 Antibody With Selected Fc Receptors
Source: Front Chem. 2021 Jul 2;9:705931. doi: 10.3389/fchem.2021.705931 (PMC8283507; doi:10.3389/fchem.2021.705931)
Supplement: Supplementary file 1 [file DataSheet1.DOCX]

SUPPLEMENTARY

The model obtained with homology modeling was validated using several tools. The results and a quick description of the methods used are provided below.

Homology modeling report for reference structure 1HZH:

ERRAT

Errat tool provides the statistics of non-bonded interactions between different atom types and plots the value of the error function as a function of the position of a 9-residue sliding window, computed by comparison with statistics from highly refined structures(Colovos and Yeates, 1993).

Score: Overall Quality Factor 81.4123

VERIFY Complete

Determines the compatibility of an atomic model (3D) with its own amino acid sequence (1D) by assigning a structural class based on its location and environment (alpha, beta, loop, polar, nonpolar etc.) and comparing the results to good structures(Bowie et al., 1991).

Score:

94.54% of the residues have

averaged 3D-1D score >= 0.2

Pass

At least 80% of the amino acids have scored >= 0.2 in the 3D/1D profile

PROVE

Calculates the volumes of atoms in macromolecules using an algorithm which treats the atoms like hard spheres and calculates a statistical Z-score deviation for the model from highly resolved (2.0 Å or better) and refined (R-factor of 0.2 or better) PDB-deposited structures(Pontius et al., 1996).

Score:

PROVE Complete

Buried outlier protein atoms total, from 1 Model: 0.0%

Pass

PROCHECK

Checks the stereochemical quality of a protein structure by analyzing residue-by-residue geometry and overall structure geometry(Laskowski et al., 1993).


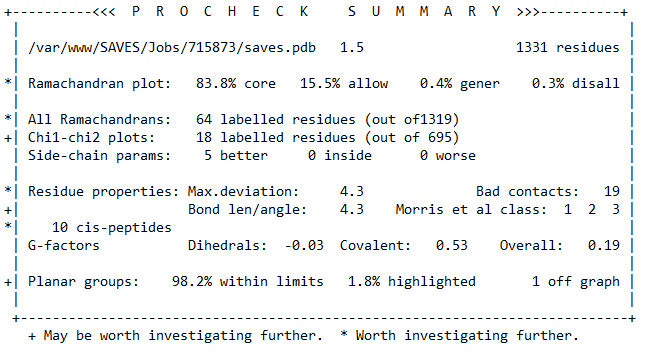


Figure S1: PROCHECK report for the 1HZH (full antibody structure)

REPORT Homology modeling report for the Homology model based on full antibody template (PDB ID: 1HZH ) and Fab region (PDB ID: 5TRU) :

ERRAT

Score: Overall quality factor 80.9756

VERIFY Complete

Score:

90.95% of the residues have

averaged 3D-1D score >= 0.2

Pass

At least 80% of the amino acids have scored >= 0.2 in the 3D/1D profile.

PROVE

Score:

Buried outlier protein atoms total, from 1 Model: 3.3%

PROCHECK


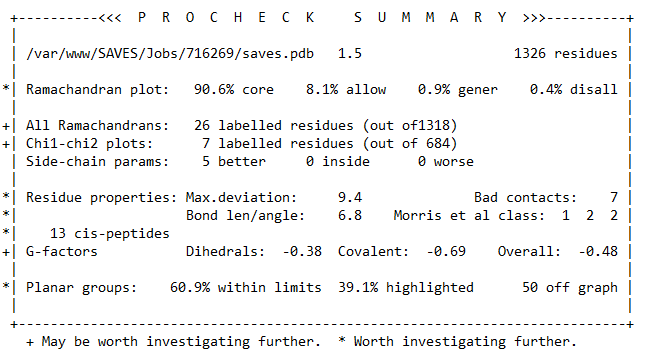


Figure S2: PROCHECK report for the modeled structure used in simulations.


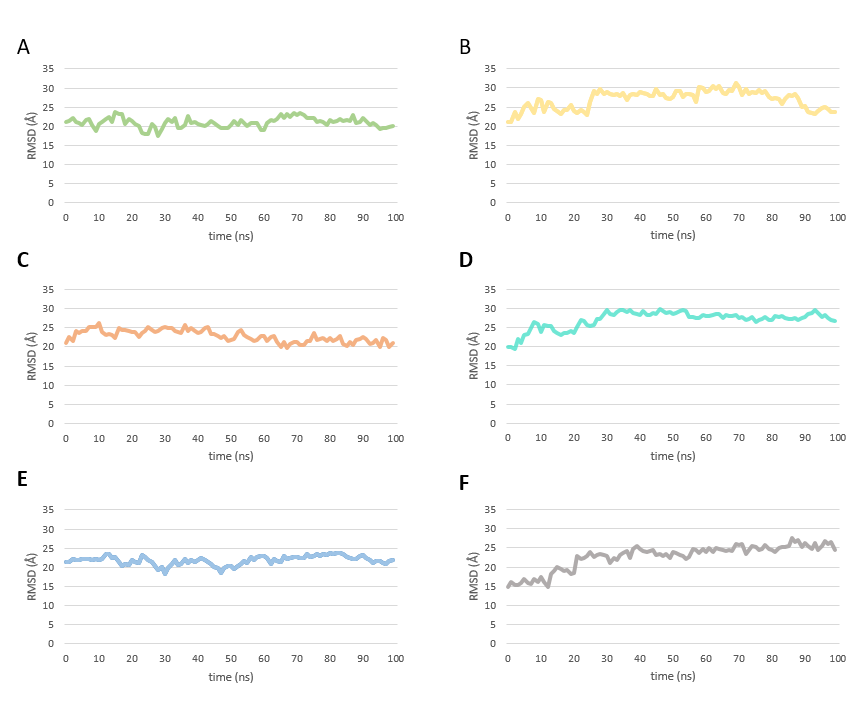


Figure S3: RMSD through time for simulations (100ns) with the whole antibody and wildtype or mutated FcγR. A) A236 antibody and FcγRIIa; B) Wildtype antibody and FcγRIIa; C) A236 antibody and FcγRIIb; D) Wildtype antibody and FcγRIIb; E) A236 antibody and FcγRIIIa; F) Wildtype antibody and FcγRIIIa.


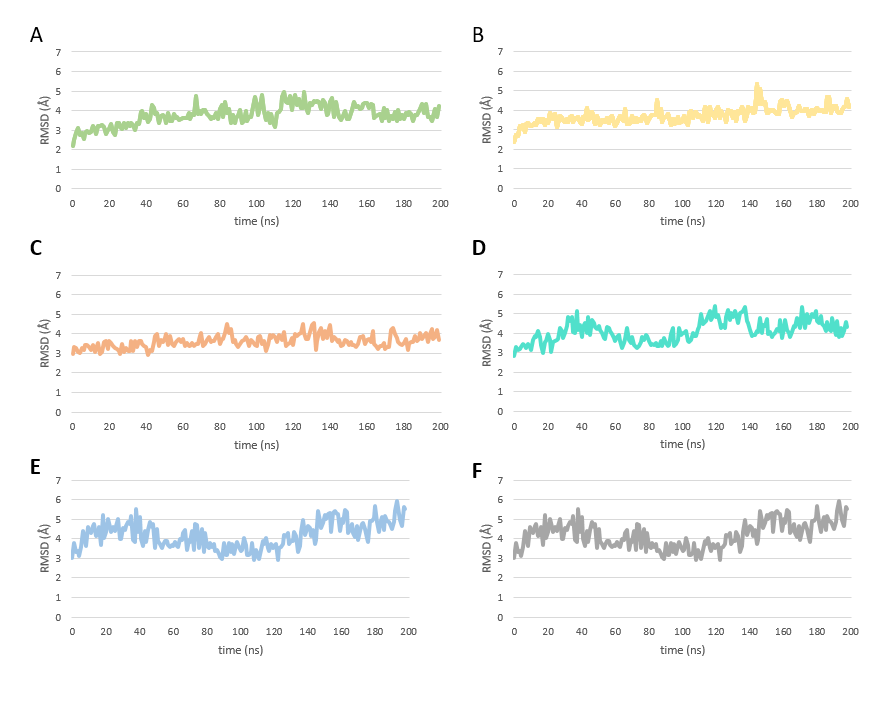


Figure S4: RMSD through time (200ns) for simulations with either the wildtype or mutated Fc antibody region and FcγR. A) A236 antibody and FcγRIIa; B) Wildtype antibody and FcγRIIa; C) A236 antibody and FcγRIIb; D) Wildtype antibody and FcγRIIb; E) A236 antibody and FcγRIIIa; F) Wildtype antibody and FcγRIIIa.


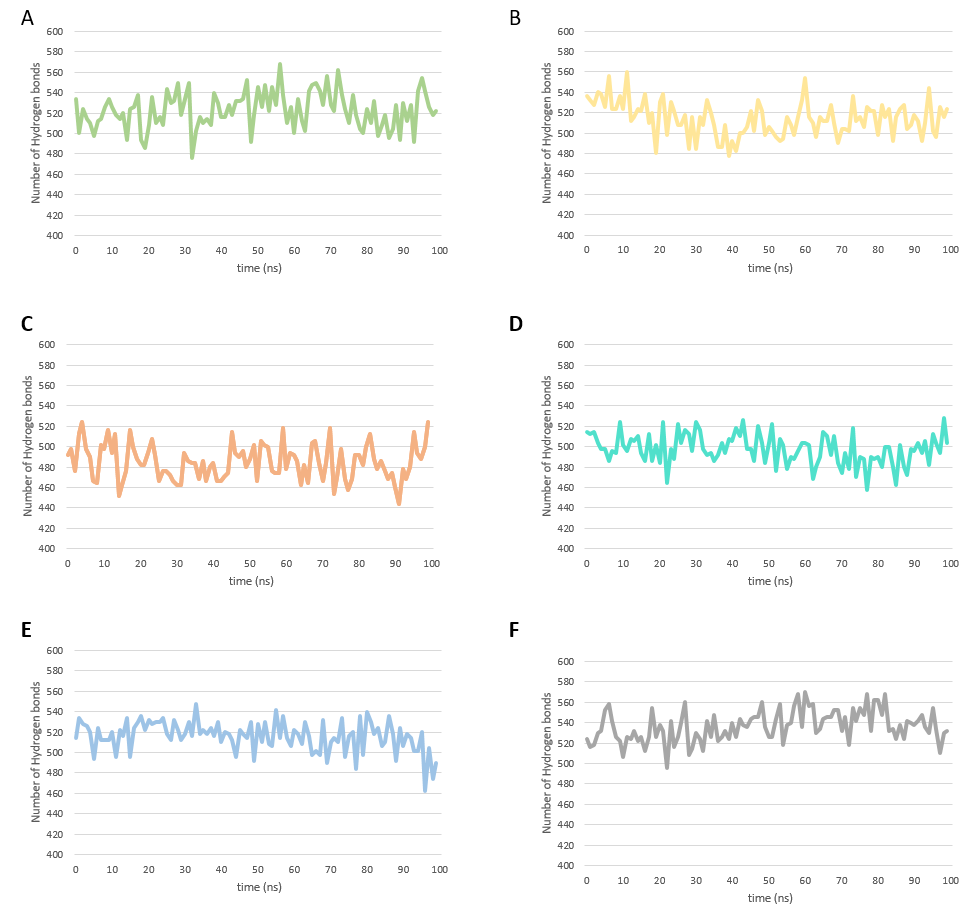


Figure S5: Hydrogen bonds through time (100ns) within the ligand( FcγR) for the whole antibody simulations: A) A236 antibody and FcγRIIa simulation; B) Wildtype antibody and FcγRIIa simulation; C) A236 antibody and FcγRIIb simulation; D) Wildtype antibody and FcγRIIb simulation; E) A236 antibody and FcγRIIIa simulation; F) Wildtype antibody and FcγRIIIa simulation.


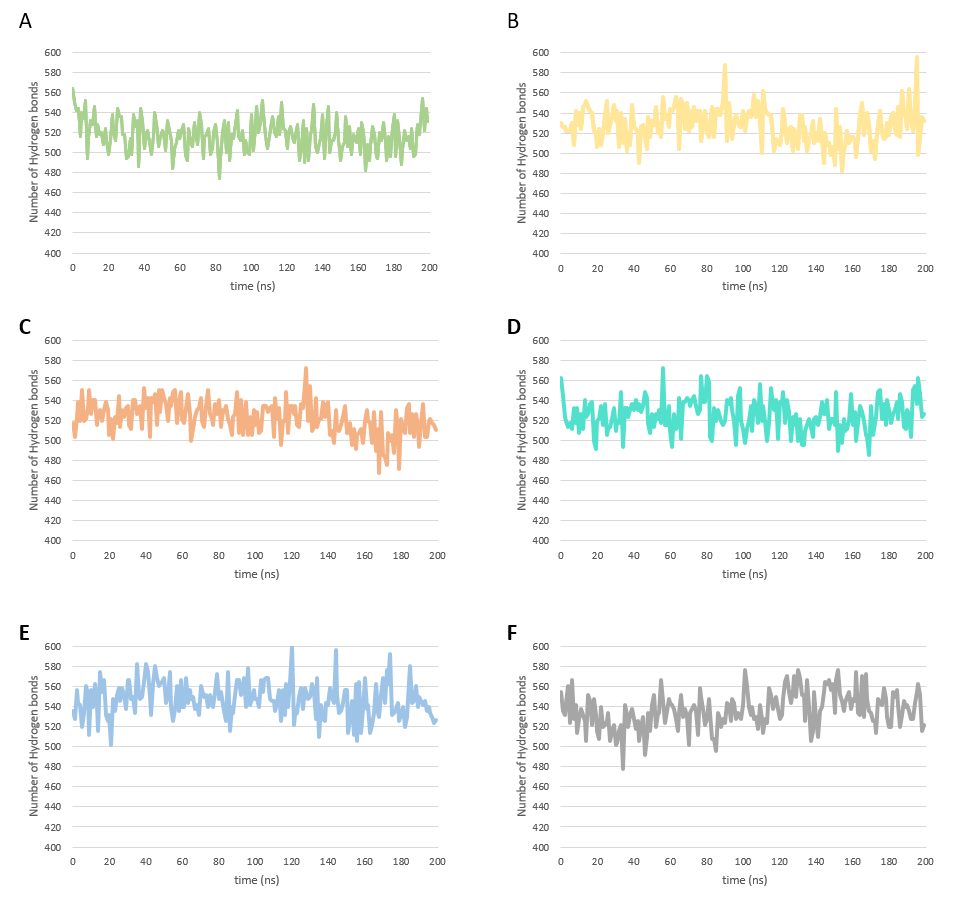


Figure S6: Hydrogen bonds through time (200ns) within the ligand (FcγR) for the Fc region simulations: A) A236 antibody and FcγRIIa simulation; B) Wildtype antibody and FcγRIIa simulation; C) A236 antibody and FcγRIIb simulation; D) Wildtype antibody and FcγRIIb simulation; E) A236 antibody and FcγRIIIa simulation; F) Wildtype antibody and FcγRIIIa simulation.


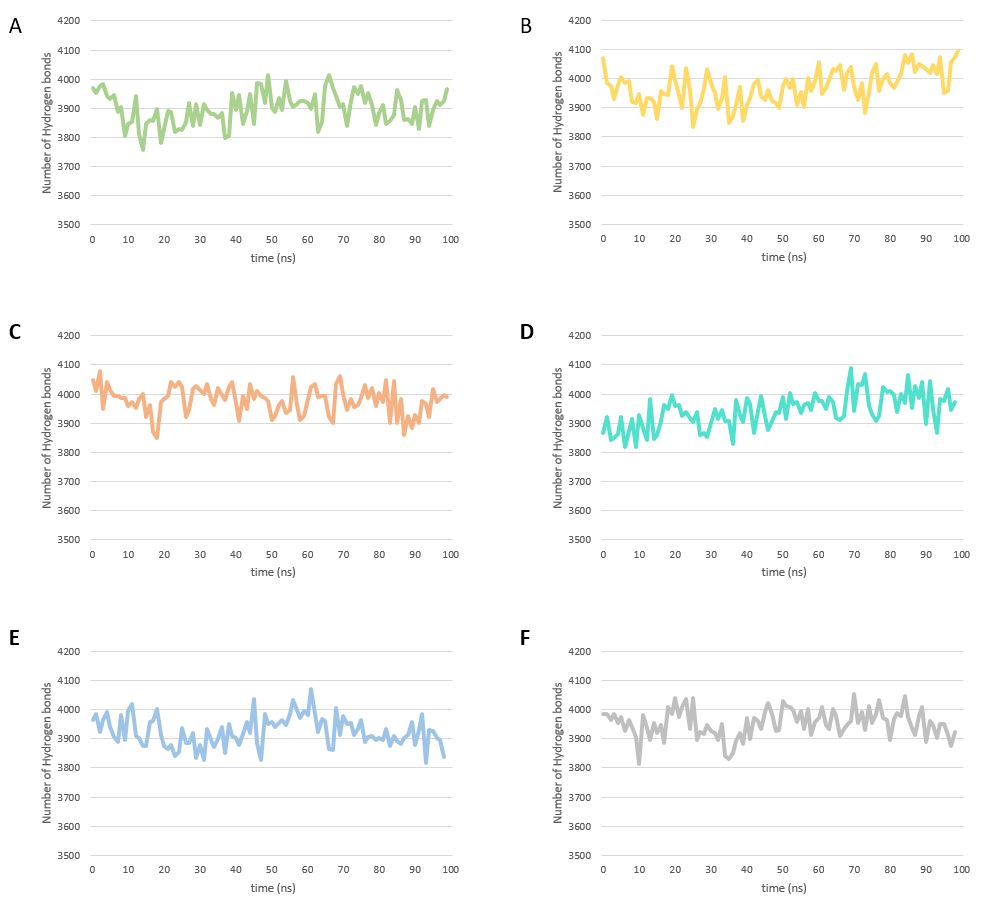


Figure S7: Hydrogen bonds through time (100ns) within the protein (full antibody): A) A236 antibody and FcγRIIa simulation; B) Wildtype antibody and FcγRIIa simulation; C) A236 antibody and FcγRIIb simulation; D) Wildtype antibody and FcγRIIb simulation; E) A236 antibody and FcγRIIIa simulation; F) Wildtype antibody and FcγRIIIa simulation.


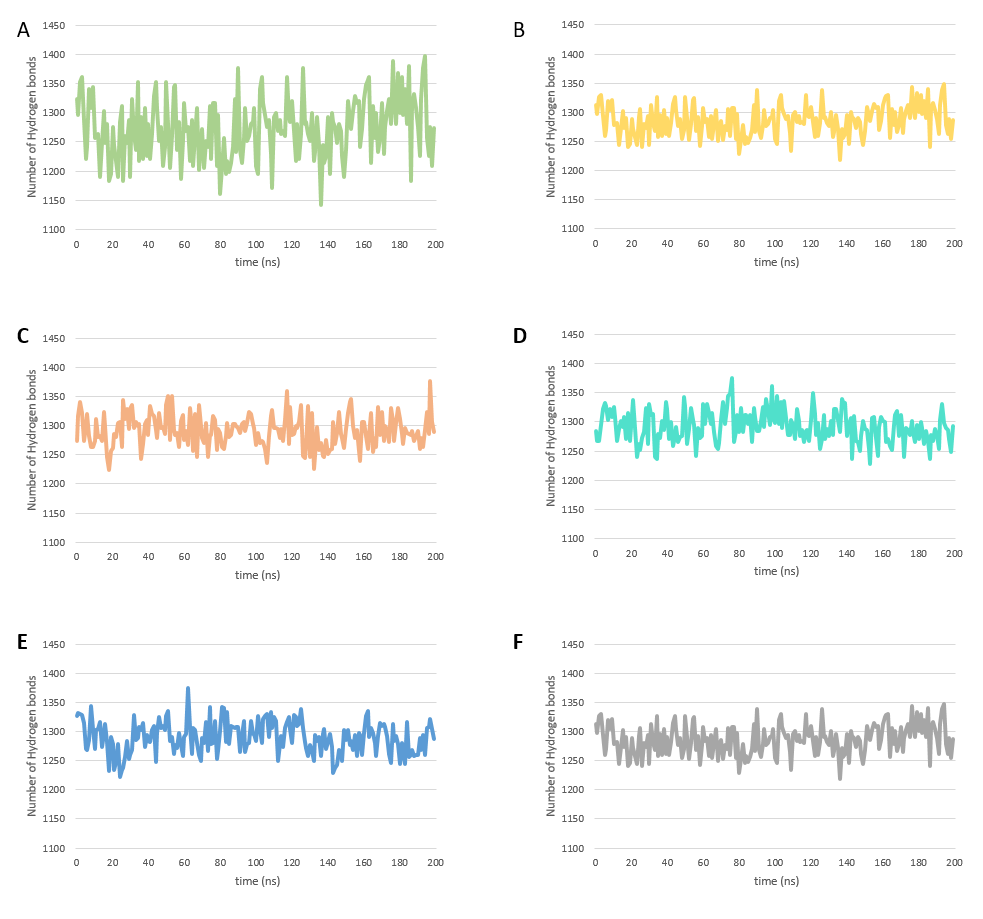


Figure S8: Hydrogen bonds through time (200ns) within the protein (antibody fwc): A) A236 antibody and FcγRIIa simulation; B) Wildtype antibody and FcγRIIa simulation; C) A236 antibody and FcγRIIb simulation; D) Wildtype antibody and FcγRIIb simulation; E) A236 antibody and FcγRIIIa simulation; F) Wildtype antibody and FcγRIIIa simulation.


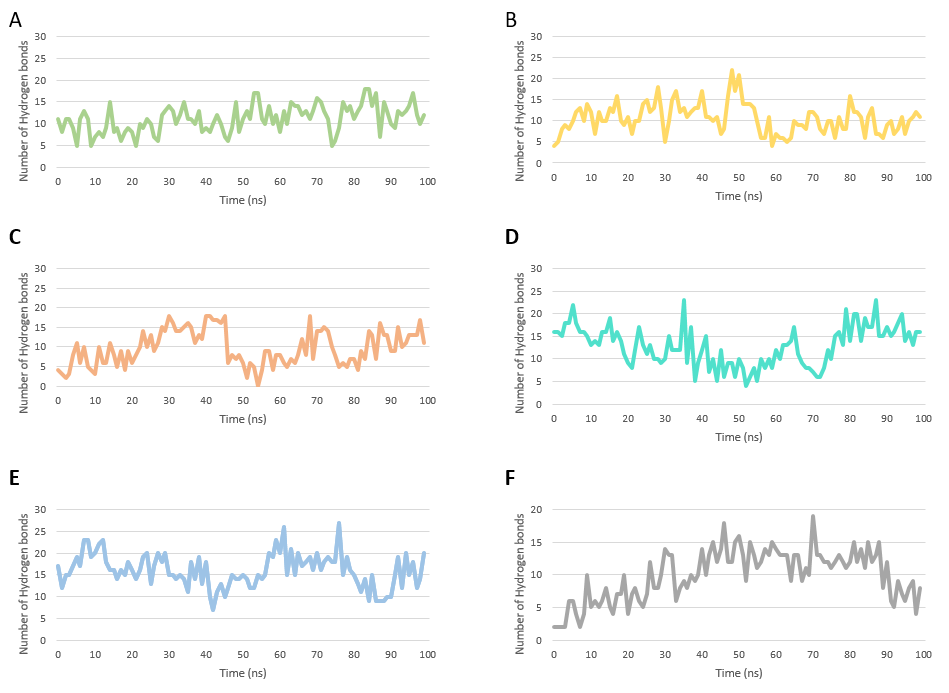


Figure S9: Hydrogen bonds through time (100ns) between the full antibody and FcγR: A) A236 antibody and FcγRIIa simulation; B) Wildtype antibody and FcγRIIa simulation ; C) A236 antibody and FcγRIIb simulation; D) Wildtype antibody and FcγRIIb simulation; E) A236 antibody and FcγRIIIa simulation; F) Wildtype antibody and FcγRIIIa simulation.


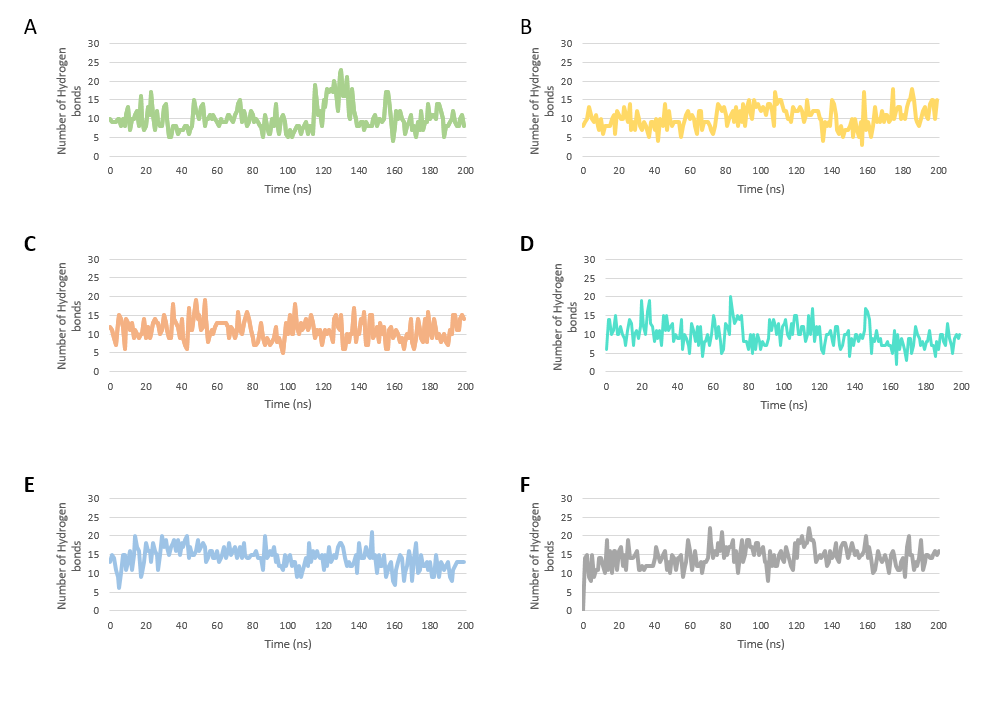


Figure S10: Hydrogen bonds through time (200ns) between the Fc region of the antibody and FcγR: A) A236 antibody and FcγRIIa simulation; B) Wildtype antibody and FcγRIIa simulation ; C) A236 antibody and FcγRIIb simulation; D) Wildtype antibody and FcγRIIb simulation; E) A236 antibody and FcγRIIIa simulation; F) Wildtype antibody and FcγRIIIa simulation.

Table S1: Average time of contact and number of contacts for the Fc regions with FcγR simulations.

| Simulation (Fc region- FcγR) | Average time of contact(ps) | Average number of contacts/ns |
| --- | --- | --- |
| **Fcγ2a A236** | 12 | 26,1 |
| **Fcγ2b A236** | 11,84 | 17,2 |
| **Fcγ3a A236** | 11,79 | 9,8 |
| **Fcγ2a wt** | 12,7 | 24,3 |
| **Fcγ2b wt** | 11,11 | 12 |
| **Fcγ3a wt** | 11.19 | 20,5 |

Table S2: Average time of contact and number of contacts for the Full antibody with FcγR simulations.

| Simulation (Full ab- FcγR) | Average time of contact(ps) | Average number of contacts/ns |
| --- | --- | --- |
| **Fcγ2a A236** | 11,49 | 3,1 |
| **Fcγ2b A236** | 12,06 | 5,2 |
| **Fcγ3a A236** | 13,21 | 10,6 |
| **Fcγ2a wt** | 11,43 | 2,2 |
| **Fcγ2b wt** | 11,42 | 3,8 |
| **Fcγ3a wt** | 11,23 | 8,2 |

SUPPLEMENTARY CITATIONS

Bowie, J., Luthy, R., and Eisenberg, D. (1991). A method to identify protein sequences that fold into a known three-dimensional structure. *Science* 253, 164–170. doi:10.1126/science.1853201.

Colovos, C., and Yeates, T. O. (1993). Verification of protein structures: patterns of nonbonded atomic interactions. *Protein Sci* 2, 1511–1519. doi:10.1002/pro.5560020916.

Laskowski, R. A., MacArthur, M. W., Moss, D. S., and Thornton, J. M. (1993). PROCHECK: a program to check the stereochemical quality of protein structures. *J Appl Crystallogr* 26, 283–291. doi:10.1107/S0021889892009944.

Pontius, J., Richelle, J., and Wodak, S. J. (1996). Deviations from standard atomic volumes as a quality measure for protein crystal structures. *J Mol Biol* 264, 121–136. doi:10.1006/jmbi.1996.0628.

Yogo, R., Yamaguchi, Y., Watanabe, H., Yagi, H., Satoh, T., Nakanishi, M., et al. (2019). The Fab portion of immunoglobulin G contributes to its binding to Fcγ receptor III. *Sci Rep* 9, 11957. doi:10.1038/s41598-019-48323-w.
